# Development of an oncogenic dedifferentiation SOX signature with prognostic significance in hepatocellular carcinoma

**DOI:** 10.1186/s12885-019-6041-2

**Published:** 2019-08-28

**Authors:** Mei-Mei Li, Yun-Qiang Tang, Yuan-Feng Gong, Wei Cheng, Hao-Long Li, Fan-En Kong, Wen-Jie Zhu, Shan-Shan Liu, Li Huang, Xin-Yuan Guan, Ning-Fang Ma, Ming Liu

**Affiliations:** 1 Affiliated Cancer Hospital & Institute of Guangzhou Medical University, Key Laboratory of Protein Modification and Degradation, School of Basic Medical Sciences, Guangzhou Medical University, Guangzhou, China; 2 State Key Laboratory of Respiratory Disease, Guangzhou Medical University, Guangzhou, China; 3 Department of Clinical Oncology, State Key Laboratory for Liver Research, The University of Hong Kong, Pok Fu Lam, Hong Kong

**Keywords:** Oncogenic dedifferentiation, Prognostic value, Stem cell-like properties

## Abstract

**Background:**

Gradual loss of terminal differentiation markers and gain of stem cell-like properties is a major hall mark of cancer malignant progression. The stem cell pluripotent transcriptional factor SOX family play critical roles in governing tumor plasticity and lineage specification. This study aims to establish a novel SOX signature to monitor the extent of tumor dedifferentiation and predict prognostic significance in hepatocellular carcinoma (HCC).

**Methods:**

The RNA-seq data from The Cancer Genome Atlas (TCGA) LIHC project were chronologically divided into the training (*n* = 188) and testing cohort (*n* = 189). LIRI-JP project from International Cancer Genome Consortium (ICGC) data portal was used as an independent validation cohort (*n* = 232). Kaplan-Meier and multivariable Cox analyses were used to examine the clinical significance and prognostic value of the signature genes.

**Results:**

The SOX gene family members were found to be aberrantly expressed in clinical HCC patients. A five-gene SOX signature with prognostic value was established in the training cohort. The SOX signature genes were found to be closely associated with tumor grade and tumor stage. Liver cancer dedifferentiation markers (AFP, CD133, EPCAM, and KRT19) were found to be progressively increased while hepatocyte terminal differentiation markers (ALB, G6PC, CYP3A4, and HNF4A) were progressively decreased from HCC patients with low SOX signature scores to patients with high SOX signature scores. Kaplan-Meier survival analysis further indicated that the newly established SOX signature could robustly predict patient overall survival in both training, testing, and independent validation cohort.

**Conclusions:**

An oncogenic dedifferentiation SOX signature presents a great potential in predicting prognostic significance in HCC, and might provide novel biomarkers for precision oncology further in the clinic.

**Electronic supplementary material:**

The online version of this article (10.1186/s12885-019-6041-2) contains supplementary material, which is available to authorized users.

## Background

Liver cancer ranks the fifth most prevalent cancers in the world and the second leading cause of cancer death. Lack of suitable biomarkers for early detection and limited treatment strategies are the major causes of high mortality [1]. Although it’s still under debate whether cancer originates from embryonic stem cells or undergoes dedifferentiation from terminally differentiated cells, the critical roles of developmental signaling pathways in cancer initiation and malignant progression have been widely accepted [2, 3]. Increasing evidences suggested that critical molecules which regulate embryonic stem cell pluripotency and differentiation are usually activated in the tumor tissue [4–6]. Aberrant activation of those developmental networks can also induce retro-differentiation or trans-differentiation between different cellular lineages including liver progenitors, hepatocytes, and cholangiocytes, which constitute the cellular heterogeneity of liver cancer [7–9]. Monitoring the extent of tumor dedifferentiation and patient prognosis might help define different subgroups of patients for precision treatment. However, effective biomarkers are still lacking for clinical use.

The Sox (Sry-related high-mobility groupbox) family of transcription factors have been well appreciated in multiple aspects of development including sex determination, embryogenesis, organogenesis, neurogenesis, skeletogenesis and hematopoiesis [10, 11]. SOX proteins are functionally divided into 9 subgroups termed A to H according to the degree of similarity of their HMG-box amino acids and flanking regions: Subgroup A (SRY), Subgroup B1 (SOX1, SOX2 and SOX3), Subgroup B2 (SOX14 and SOX21), Subgroup C (SOX4, SOX11 and SOX12), Subgroup D (SOX5, SOX6 and SOX13), Subgroup E (SOX8, SOX9 and SOX10), Subgroup F (SOX7, SOX17 and SOX18), Subgroup G (SOX15) and Subgroup H (SOX30) [12–14]. Beyond the functions of well-established regulators of development, growing evidences have linked SOX families with human diseases, particularly in tumors. SOX family members were shown to mastermind the tumor initiating potential of cancer cells in driving cancer pluripotent stem cells establishment, stem cell maintenance, and lineage fate determinant in various types of cancers [15–20]. In the present study, we established a novel oncogenic dedifferentiation SOX signature to effectively monitor the extent of tumor dedifferentiation and predict patient prognosis in HCC. Further incorporation of the gene signature into clinical RNA-seq profiling might help identify groups of high-risk patients for precision medicine.

## Methods

### Clinical cohort and RNA-seq data sets

We obtained RNA-seq mRNA expression data and clinical pathological data of liver cancer from the LIHC project of TCGA (https://tcgadata.nci.nih.gov/tcga/). The data was downloaded using the University of California Santa Cruz cancer genomics data portal UCSC Xena (https://xena.ucsc.edu/). The LIHC project contains 50 normal liver tissue samples and 377 primary liver cancer tissue samples. Samples from TCGA data set were divided chronologically into training (TCGA-LIHC Cohort I, *n* = 188) and testing cohorts (TCGA-LIHC Cohort II, *n* = 189), and we did not find any bias in TCGA test and validation set in case bias analysis. A total of 232 samples with RNA-Seq mRNA expression data and clinical pathological data were obtained from the ICGC portal (https://dcc.icgc.org/projects/LIRI-JP) as an independent validation cohort. These samples belong to a Japanese population primarily infected with HBV/HCV [21]. We used the normalized read count values given in the gene expression file. Detailed clinical background information of the patients could be found in Additional file 1: Table S1. Studies using human tissues were reviewed and approved by the Committees for Ethical Review of Research involving Human Subjects of Guangzhou Medical University. The studies were conducted in accordance with International Ethical Guidelines for Biomedical Research Involving Human Subjects (CIOMS). All patients gave written informed consent for the use of their clinical specimens for medical research.

### Statistical analysis and signature score generation

The differential expression profiles between tumor tissues and the normal liver tissues were generated based on the normalized expression value of RNA-seq data. Independent student’s t test was used to compare the mean expression level of two different groups. One-way ANOVA test was used to compare means between 3 and more subgroups. The test was performed in GraphPad Prism 5 (La Jolla, CA, USA). Kaplan–Meier survival curves of the two risk groups were plotted and the log-rank *P* value of the survival difference calculated between them. The association of SOX signature subgroups with clinical features was examined by Pearson’s χ^2^ test. Univariate and multivariable Cox proportional hazards regression was used to assess association with overall survival using SPSS v19 (IBM, Inc., Chicago, IL, USA). P value less than 0.05 was considered statistically significant. The oncogenic dedifferentiation SOX signature was generated by taking into account the expression of individual sox family genes and their clinical association with patient overall survival time. A SOX signature score was calculated according to the expression of each signature gene. HCC patient with overexpression (defined as the normalized expression value above median in the tumor tissues) of each sox signature gene will be given “1” score. The sum of the 5 SOX signature genes (SOX3, SOX4, SOX11, SOX12, SOX14) forms the final SOX signature score. Patients with SOX signature score value greater than 2 was defined as “High SOX signature group”, and with score value less than and including 2 was defined as “Low SOX signature group”. The cBio Cancer Genomics Portal was used to establish a network connection of SOX signature targets and other closely associated genes [22, 23]. Gene ontology analysis and signaling pathway analysis was performed using DAVID Bioinformatics Resources [24, 25].

### RNA extraction and quantitative real-time PCR

Total RNA was extracted using TRIZOL Reagent (Life technologies, Carlsbad, CA), and reverse transcription was performed using an Advantage RT-for-PCR Kit (Clontech Laboratories, Mountain View, CA) according the manufacturer’s instructions. For qPCR analysis, aliquots of double-stranded cDNA were amplified using a SYBR Green PCR Kit (Life technologies, Carlsbad, CA) and an ABI PRISM 7900 Sequence Detector. Sequences of primers used in this study were listed in Additional file 2: Table S2. For cell lines, the relative gene expression is given as 2^−ΔCT^ (ΔCT = CT (gene) – CT (18S)) and normalized to the relative expression that was detected in the corresponding control cells. For clinical samples, we calculated the relative expressions of target genes in clinical HCCs and their matched nontumor specimens by the formula 2^−ΔCT^ (ΔCT = CT (target genes) – CT (18S)) and normalized to the average relative expression in all of the nontumor tissues, which was defined as 1.0.

### Immunohistochemical staining (IHC)

Paraffin-embedded tissue sections were deparaffinized and rehydrated. Slides were immersed in 10 mM citrate buffer and boiled for 15 min in microwave oven and then incubated with primary antibody at 4 °C overnight in a moist chamber and then sequentially incubated with biotinylated general secondary antibody for 1 h at room temperature, streptavidin-peroxidase conjugate for 15 min at room temperature. Finally, the 3, 5-diaminobenzidine (DAB) Substrate Kit (Dako, Carpinteria, CA) was used for color development followed by Mayer’s hematoxylin counterstaining.

## Results

### Compiling a biology-based prognostic dedifferentiation SOX gene signature in HCC

**Fig. 1:**
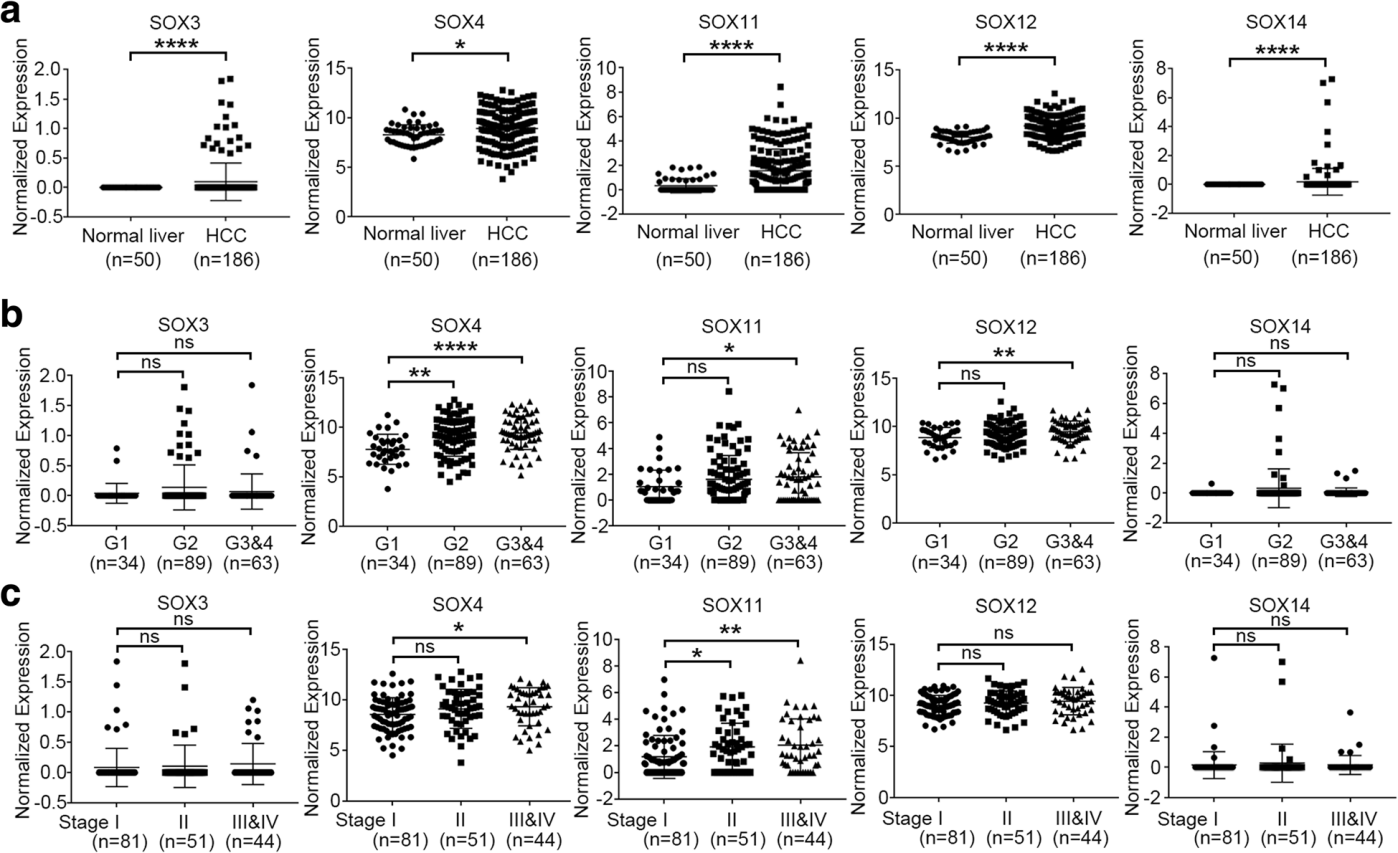
Expression of SOX signature genes in HCC patients. **a** The normalized expression of SOX signature genes (SOX3, SOX4, SOX11, SOX12, and SOX14) were compared between 50 normal liver tissues and 186 HCC tissues from the TCGA-LIHC Cohort I. **b** The normalized expressions of SOX signature genes were compared between HCC patient subgroups with different tumor grade. **c** The normalized expressions of SOX signature genes were compared between HCC patient subgroups with different tumor stage. Independent student’s t test, *, *P* < 0.05, **, *P* < 0.01, ***, *P* < 0.001, ****, *P* < 0.0001, ns, not significant. The figures were generated using GraphPad Prism 5

Considering the important roles of the SOX gene family in regulating stem cell pluripotency, tumor cell plasticity and differentiation, we tried to establish a SOX gene signature to monitor tumor differentiation and stratify patient overall survival in HCC. To comprehensively analyze the expression profile and prognostic significance of SOX family members in HCC, The Cancer Genome Atlas (TCGA) hepatocellular carcinoma cohort was divided chronologically into a training cohort (TCGA-LIHC Cohort I, *n* = 188) and a validation cohort (TCGA-LIHC Cohort II, *n* = 189). The mRNA expression data and clinical data were downloaded using the UCSC XENA portal. The demographics of these cohorts were well balanced, and the clinical pathological information was shown in Additional file 1: Table S1. The relative expression of all 19 SOX family members excluding SRY, which was absently expressed in both liver and HCC tissues, was compared in the 188 HCC cases from TCGA-LIHC Cohort I and 50 normal liver tissues from TCGA-LIHC project. Most of the SOX family members were found to be aberrantly expressed in HCC. SOX2, SOX3, SOX4, SOX11, SOX12, SOX13, SOX14, SOX18, and SOX21 were found to be significantly up-regulated in HCC. SOX5, SOX6, SOX7, and SOX10 were found to be significantly down-regulated in HCC (Table 1). Kaplan–Meier survival analysis showed that SOX3, SOX4, SOX11, SOX12, SOX14, and SOX17 were significantly associated with patient overall survival (Table 1). Taken together, SOX3, SOX4, SOX11, SOX12, and SOX14 were aberrantly expressed in HCC with prognostic significance, and were selected as SOX signature genes for further validation (Fig. 1a). The significant up-regulation of the SOX signature genes were further confirmed by qPCR in 21 paired HCC clinical samples (Additional file 3: Figure S1). Overexpression of the representative SOX signature gene SOX11 was also found in paired HCC tissues by IHC staining (Additional file 4: Figure S2).

**Table 1:** Relative expression and prognosis of sox family genes in the training cohort (TCGA-LIHC cohort I, n = 188)

Gene	Expression				Overall survival		
	Mean normalized expression		Trend	P Value^a^	Mean OS time (months)		P Value^#^
	HCC	Normal liver			Low expression	High expression	
SOX1	0.2995	0.1106	Up	0.2784	996	816	0.741
SOX2	2.0960	0.7323	Up	0.0001	1017	915	0.704
SOX3	0.0967	0	Up	0.0001	1019	493	0.000
SOX4	8.9210	8.2760	Up	0.0172	1109	802	0.005
SOX5	5.8270	7.1320	Down	0.0001	913	1012	0.801
SOX6	6.6730	8.7640	Down	0.0001	1027	906	0.747
SOX7	6.4660	7.3970	Down	0.0001	906	976	0.267
SOX8	2.3920	2.3090	Up	0.7006	895	1019	0.225
SOX9	8.7280	8.2970	Up	0.2150	995	928	0.609
SOX10	0.8675	2.5260	Down	0.0001	1055	861	0.092
SOX11	1.5660	0.3328	Up	0.0001	1120	790	0.001
SOX12	9.2010	8.0270	Up	0.0001	1017	910	0.010
SOX13	10.2500	9.4920	Up	0.0001	971	924	0.138
SOX14	0.1806	0	Up	0.0001	1002	630	0.019
SOX15	2.7610	2.5210	Up	0.0562	930	996	0.261
SOX17	5.7740	5.6750	Up	0.5462	859	1054	0.019
SOX18	7.8070	7.1120	Up	0.0001	930	963	0.121
SOX21	0.8787	0.1807	Up	0.0019	1013	883	0.059
SOX30	0.8157	0.7922	Up	0.8675	929	974	0.692

^a^, Unpaired student t test

^#^, Kaplan Meier survival Log-rank P value

### The SOX signature represents an oncogenic dedifferentiation phenotype

**Fig. 2:**
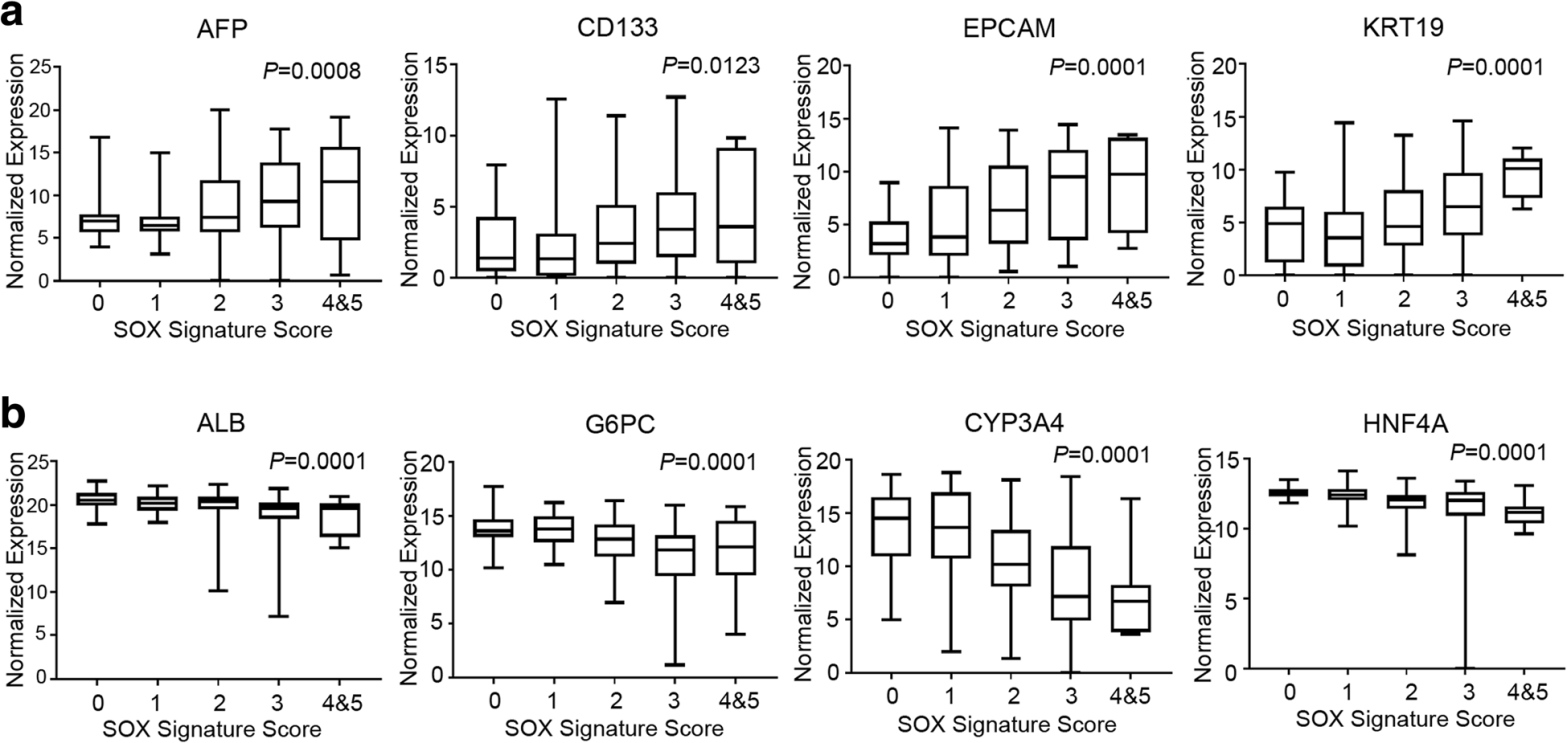
The SOX signature represents an oncogenic dedifferentiation phenotype. **a** The normalized expressions of liver cancer dedifferentiation markers and liver progenitor cell markers in HCC patients with different SOX signature score. **b** The normalized expressions of hepatocyte terminal differentiation markers in HCC patients with different SOX signature score. One-way ANOVA test. *P* value less than 0.05 was considered statistically significant. The figures were generated using GraphPad Prism 5

In clinical pathology, tumor grade represents the extent of how tumor tissues resemble their normal counterparts. High grade tumors usually show oncogenic dedifferentiation phenotypes. The expression of SOX signature genes was examined in subgroups of patients with different tumor grade. A progressive increase of SOX signature genes could be found from low grade HCC patients to high grade HCC patients (Fig. 1b). In addition, the expression of SOX signature genes also progressively increases from early stage HCC patients to late stage HCC patients (Fig. 1c). Poorly differentiated tumors usually indicate the activation of cancer stem cells or progenitor cells. This process is accompanied with increase of stem cell markers, and decrease of terminal differentiation markers. We further established a score system to quantitatively define the SOX signature in HCC patients. Patient with overexpression (defined as the normalized expression value above median level in the tumor tissues) of each sox signature gene will be given “1” score, and the sum of the 5 SOX signature genes forms the final SOX signature score. We examined the liver cancer stem cell or progenitor markers (AFP, CD133, EPCAM, and KRT19), and hepatocyte terminal differentiation markers (ALB, G6PC, CYP3A4, and HNF4A) in subgroup of patients with different SOX signature scores. A significant positive correlation of liver cancer stem cell or progenitor markers, and a significant negative correlation of hepatocyte terminal differentiation markers with SOX signature scores could be found in the HCC patients (Fig. 2a and b). These findings indicated that the SOX signature represents an oncogenic dedifferentiation phenotype, and is activated in high grade and late stage tumors.

### Prediction of the SOX signature-regulated transcriptional network

**Fig. 3:**
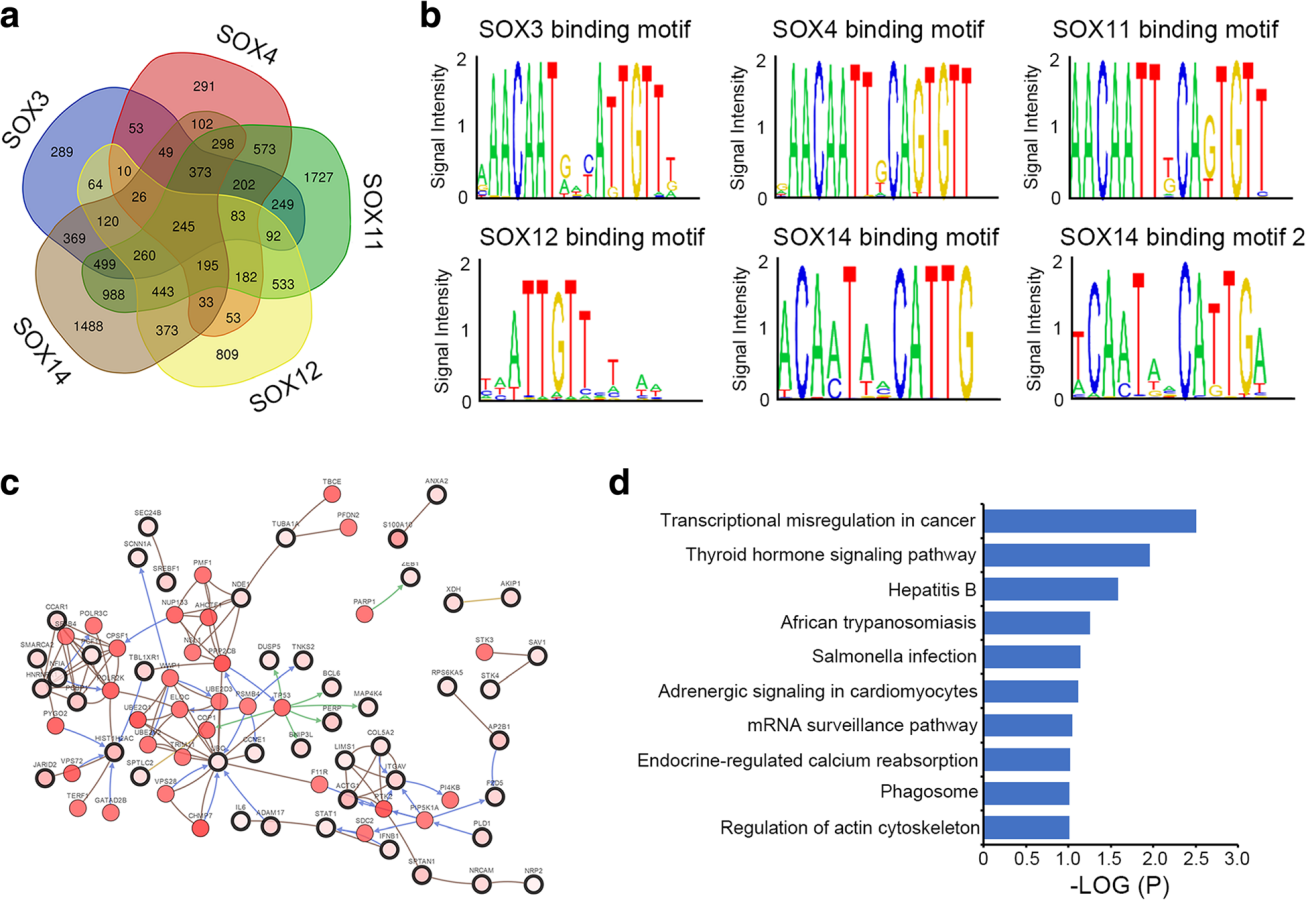
Prediction of the SOX signature-regulated transcriptional network. **a** The Venn diagram show overlapping downstream targets of SOX signature genes. **b** Prediction of SOX signature gene binding motif. **c** Network of SOX signature gene downstream targets and their associated genes. **d** Gene ontology and signaling pathway analysis of SOX signature gene downstream targets

Considering the SOX family members are transcriptional factors that regulate gene expression, the binding motifs and downstream targets of SOX signature genes were predicted using a systems genetics approach [26]. The common downstream targets of the five SOX signature genes were plotted using the online Venn diagram tool (http://bioinformatics.psb.ugent.be/webtools/Venn/). A total of 245 genes were found to be commonly regulated by the SOX signature (Fig. 3a, Additional file 5: Table S3). High-frequency binding motifs of each SOX signature genes were also predicted (Fig. 3b). The downstream targets of SOX signature genes formed a comprehensive network, which closely associated with critical transcriptional regulators of embryonic development including TP53, ZEB1, SMARCA2, and JARID2 (Fig. 3c). Gene ontology analysis also revealed the signaling pathways significantly associated with SOX signature target genes (Fig. 3d).

### The association of SOX signature with clinical pathological features in HCC

To investigate the clinical significance of SOX signature, the patients were further classified into two subgroups. The “High sox signature group” was defined with a sox signature score greater than 2, and the “Low SOX signature group” was defined with a sox signature score less than and including 2. The association of the SOX signature with clinical pathological features were examined by Pearson’s χ^2^ test in the TCGA-LIHC Cohort I (Table 2). The five-gene SOX signature was further tested in two independent clinical cohorts for validation using the same risk score threshold chosen in the TCGA-LIHC cohort I. The association of the SOX signature with clinical pathological features were also examined by Pearson’s χ^2^ test in the TCGA-LIHC Cohort II and the LIRI-JP Cohort (Table 2).

**Table 2:** Clinical pathological features of sox signature genes in three cohorts

	TCGA LIHC Cohort I (n = 188)			TCGA LIHC Cohort II (n = 189)			LIRI-JP Cohort (*n* = 231)		
	Low sox group	High sox group	P value	Low sox group	High sox group	P value	Low sox group	High sox group	P value
Gender			0.020			0.014			0.671
Male	104 (55.3%)	28 (14.9%)		100 (52.9%)	23 (12.2%)		141 (61.0%)	30 (13.0%)	
Female	35 (18.6%)	21 (11.2%)		43 (22.8%)	23 (12.2%)		48 (20.8%)	12 (5.2%)	
Tumor Stage			0.001			0.009			0.055
I	70 (37.2%)	11 (5.9%)		80 (42.3%)	14 (7.4%)		31 (13.4%)	4 (1.7%)	
II	33 (17.6%)	18 (9.6%)		28 (14.8%)	8 (4.2%)		91 (39.4%)	15 (6.5%)	
III	24 (12.8%)	19 (10.1%)		26 (13.8%)	18 (9.5%)		55 (23.8%)	16 (6.9%)	
IV	1 (0.5%)	0 (0%)		2 (1.1%)	1 (0.5%)		12 (5.2%)	7 (3.1%)	
Tumor Grade			0.026			0.126			NA
G1	32 (17.0%)	2 (1.1%)		18 (9.5%)	3 (16.7%)		NA	NA	
G2	62 (33.0%)	27 (14.4%)		73 (38.6%)	18 (9.5%)		NA	NA	
G3	37 (19.7%)	19 (10.1%)		45 (23.8%)	23 (12.2%)		NA	NA	
G4	6 (3.2%)	1 (0.5%)		5 (2.6%)	1 (0.5%)		NA	NA	
Vascular Invasion			0.003			0.001			NA
Macro	3 (1.6%)	2 (1.1%)		4 (2.1%)	8 (4.2%)		NA	NA	
Micro	41 (21.8%)	19 (10.1%)		27 (14.3%)	7 (3.7%)		NA	NA	
None	79 (42.0%)	14 (7.4%)		96 (50.8%)	21 (11.1%)		NA	NA	

### The relation between the SOX signature and the prognosis of HCC patients

**Fig. 4:**
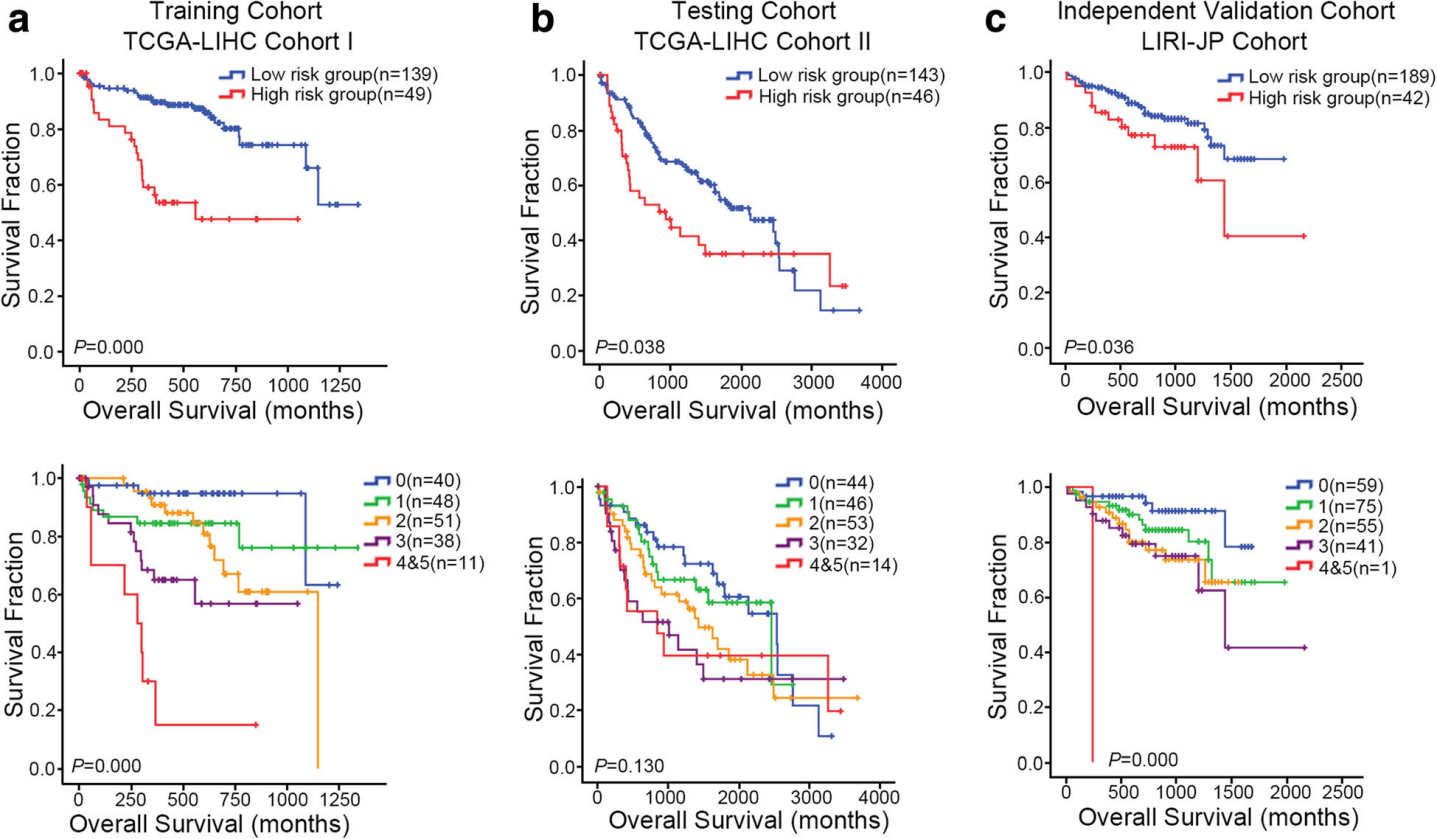
The prognostic significance of SOX signature genes in multiple HCC clinical cohorts. **a** The patients in the training set (TCGA-LIHC Cohort I, *n* = 188) were divided into “High sox group” and “Low sox group” according to the SOX signature score. Kaplan–Meier survival curves of the two risk groups were plotted and the log-rank P value of the survival difference calculated between them (Upper panel). Kaplan–Meier survival curves of HCC patients from subgroups with different SOX signature score (Lower panel). **b** Similar analysis was down in the testing set (TCGA-LIHC Cohort II, *n* = 189). **c** and validated in an independent validation set (LIRI-JP Cohort, *n* = 232). P value less than 0.05 was considered statistically significant. The figures were generated using SPSS v19

Kaplan–Meier survival analysis showed that the “High SOX signature group” had significantly worse overall survival than the “Low sox signature group” in the TCGA-LIHC Cohort I (HR = 4.045, 95% CI = 2.174–7.525, *P* = 0.000). The progressive decrease in mean survival time could also be found when the curves were plotted according to different sox signature scores (Fig. 4a). The SOX signature significantly stratified the TCGA-LIHC cohort II for overall survival (HR = 1.618, 95% CI = 1.023–2.560, *P* = 0.040) (Fig. 4b, Table 3). In a second independent LIRI-JP Cohort, again using the same risk score in the TCGA-LIHC cohort I, the SOX signature was also able to significantly stratified patients for overall survival (HR = 2.012, 95% CI = 1.031–3.926, *P* = 0.041) (Fig. 4c). In addition, Cox proportional hazards regression analysis further indicated the SOX signature as a promising predictor of patient overall survival both in the univariate overall survival analysis (Table 3). These results suggested that our newly established oncogenic dedifferentiation SOX signature could robustly predict HCC patient’s overall survival in multiple clinical cohorts.

**Table 3:** Univariate and multivariate overall survival analysis in 3 HCC cohorts

	Univariate Analysis			Multivariate Analysis		
	HR	95% CI	P value	HR	95% CI	P value
TCGA-LIHC Cohort I						
Gender						
Male vs. Female	1.351	0.724–2.521	0.345	1.508	0.575–3.957	0.404
Albumin (g/L)						
> =35 vs. < 35	0.400	0.185–0.867	0.020	0.227	0.088–0.586	0.002
AFP (ng/mL)						
> =25 vs. < 25	2.437	1.019–5.827	0.045	2.972	1.100–8.030	0.032
Tumor Stage						
III/IV vs. I/II	3.663	1.958–6.851	0.000	2.656	1.113–6.336	0.028
Tumor Grade						
G3/G4 vs. G1/G2	0.905	0.476–1.720	0.761	0.683	0.260–1.794	0.439
Vascular Invasion						
Yes vs. No	1.512	0.720–3.177	0.275	0.927	0.335–2.563	0.884
Sox Signature						
High vs. Low	4.045	2.174–7.525	0.000	1.272	0.397–4.075	0.686
TCGA-LIHC Cohort II						
Gender						
Male vs. Female	1.142	0.744–1.753	0.542	1.255	0.659–2.389	0.490
Albumin (g/L)						
> =35 vs. < 35	1.109	0.643–1.912	0.710	1.107	0.553–2.217	0.774
AFP (ng/mL)						
> =25 vs. < 25	1.347	0.815–2.229	0.246	0.874	0.454–1.680	0.685
Tumor Stage						
III/IV vs. I/II	1.914	1.203–3.048	0.006	1.826	1.117–2.984	0.016
Tumor Grade						
G3/G4 vs. G1/G2	1.198	0.776–1.849	0.415	1.336	0.900–1.982	0.150
Vascular Invasion						
Yes vs. No	1.282	0.773–2.127	0.336	1.297	0.654–2.572	0.457
Sox Signature						
High vs. Low	1.618	1.023–2.560	0.040	1.126	0.546–2.321	0.748
LIRI-JP Cohort						
Gender						
Male vs. Female	1.926	1.033–3.590	0.039	2.507	1.315–4.779	0.005
Tumor Stage						
III/IV vs. I/II	2.384	1.304–4.359	0.005	2.624	1.408–4.890	0.002
Sox Signature						
High vs. Low	2.012	1.031–3.926	0.041	1.799	0.915–3.537	0.089

## Discussion

Clinical observation of poorly differentiated tumors preserving lineage characteristics of their developmental precursor cells, indicated the strong link between tumor aggressiveness and embryonic developmental [27, 28]. Hepatocellular carcinoma (HCC) is one of the most common cancers in the world, with very poor prognosis and limited treatment methods [29]. Like many other tumors, HCC also gains embryonic-like properties, such as elevated expression of alpha-fetoprotein (AFP), which should only appear in fetal liver development. A subtype of HCC, which was usually characterized by molecular markers of bipotential hepatic progenitor cells such as CD133, EPCAM, and CK19, is predicted to have an extremely poor prognosis. [28] The critical transcriptional factors and their regulated signaling pathways governing lineage specification in development are reactivated in cancer cells and substantially contribute to malignant phenotypes such as tumor growth, metastasis, and resistance to chemotherapeutic drugs [30, 31]. Further targeting the oncogenic driving events according to tumor dedifferentiation status might provide novel therapeutic strategy for cancer treatment [32, 33]. However, biomarkers which effectively reflect the extent of HCC tumor dedifferentiation and predict patient’s outcome are still lacking currently.

In the present study, we developed a novel oncogenic dedifferentiation SOX signature and a score system to monitor the extent of tumor dedifferentiation in HCC. Taking into account the expression of individual SOX family genes and their clinical association with patient overall survival time, five SOX family members were selected as SOX signature genes. A progressive increase of liver cancer dedifferentiation markers was found from HCC patients with low SOX signature scores to patients with high SOX signature scores. Conversely, hepatocyte terminal differentiation markers were found to be progressively decreased. A training-testing-validation approach further proved that the SOX signature could robustly predict patients’ overall survival time. HCC patients with high SOX signature score also significantly associated with late stage tumors and vascular invasion. Although, the association of SOX signature with tumor grade didn’t reach statistical significance in the validation cohort, which might be due to limited sample size and the traditional morphological definition of tumor grade, most of the SOX signature genes were found progressively increased from low grade to high grade HCC patients. These clinical observations were in agreement with our previous experimental findings that the dedifferentiated tumor cells with stem cell-like properties are usually more aggressive, easy to metastasis, and resistant to chemotherapeutic drugs [34–36]. Previous molecular sub-classifications of liver cancer mainly focused on the genomic mutational landscapes and molecular signaling alterations of the tumors [37]. Recent data from genomic profiling enabled the proposals of different molecular clusters of HCCs according to their proliferation index, cellular origins and immune responses [38–41]. Interestingly, all the newly established classification models mentioned the evidence of a stem cell or progenitor cell-like properties of poor prognostic liver tumors. However, no previous reports mentioned the molecular biomarkers in defining the differentiation status and predict prognostic significance of those embryonic-related tumors. To date, several liver cancer stem cell markers such as CD133, EPCAM, CD44, KRT19 et al. have been identified and well characterized. However, due to the multiple hierarchy of stem cell progeny and the heterogeneity of the tumor, it’s difficult to define a tumor dedifferentiation state using a single cell surface marker. Considering the tumor dedifferentiation process is driven by transcriptional reprograming, we for the first time tried to define tumor differentiation status using a combination of pluripotent transcriptional factors instead of cell surface markers. Instead of stem cell or progenitor biomarkers, sox family are transcriptional factors that regulated a broad range of gene expression and critical cell fate determinants. The SOX family transcriptional factors are critical in embryonic stem cell pluripotency and tumor lineage plasticity [42, 43]. Liver cancer stem cell or progenitor biomarkers are usually also expressed on normal stem cells or regenerating hepatocytes, and their expression in the tumors are not necessarily up-regulated in the tumor tissues. This makes it difficult to quantify and discriminate cancer stem cells in evaluating patient prognosis. However, sox family genes are mostly expressed in embryonic stem cells and aberrant expression of SOX family members was also frequently found in HCC patients. Thus, using a combination of SOX family transcriptional factors might comprehensively represent the differentiation status of HCC patients and classify patients for precision oncology further in the clinic.

## Conclusions

HCC is one of the poorest prognostic tumors worldwide. High incidence of tumor relapse and lack of clear oncogenic drivers are the major challenges in HCC clinical treatment. The activation of cancer stem cells and their different hierarchy of progenies formed the heterogeneity of the tumor, and may account for the worse prognosis of the patients. However, biomarkers effectively represent the extent of HCC stem cell activation and tumor dedifferentiation are still lacking, which impeded the clinical subclassification of the patients for precision treatment. In the present study, we developed a novel oncogenic dedifferentiation gene signature and a score system to monitor the extent of tumor dedifferentiation in HCC. Five SOX family transcriptional factors were selected as SOX signature genes, and their expressions in HCC patients were evaluated to generate a SOX signature score. The score system well demonstrated HCC tumor differentiation status by comprehensively evaluating cancer stem cell or progenitor markers, and hepatocyte terminal differentiation markers. In addition, it also well stratified poor prognostic patients in several independent training-testing-validation cohorts. As RNA-seq based genetic subclassification is becoming important and cost-effective for clinical use, especially in cancer treatment, our newly established SOX signature score system might provide valuable tools for further precision diagnosis and treatment for HCC patients. Further profiling of HCC patients might provide individualized therapeutic strategy according to their unique sox signatures and contribute to precision oncology.

## Additional files

Additional file 1: **Table S1.** Clinical characteristics of the patients. (DOCX 24 kb)

Additional file 2: **Table S2.** Sequences of primers used in qPCR. (DOCX 22 kb)

Additional file 3: **Figure S1.** Relative expression of SOX signature genes in paired HCC clinical samples. (TIF 3233 kb)

Additional file 4: **Figure S2** Overexression of SOX 11 in paired HCC clinical tissues. (TIF 2043 kb)

Additional file 5: **Table S3** Predicted downstream targets of SOX signature genes. (DOCX 24 kb)

## Data Availability

The RNA-seq mRNA expression data and clinical pathological data of liver cancer from the LIHC project of TCGA was downloaded from the website: https://tcgadata.nci.nih.gov/tcga/. The data was downloaded using the University of California Santa Cruz cancer genomics data portal UCSC Xena (https://xena.ucsc.edu/). A total of 232 samples with RNA-Seq mRNA expression data and clinical pathological data from the ICGC portal was downloaded from the website: https://dcc.icgc.org/projects/LIRI-JP.

## Abbreviations

AFP: Alpha-fetal protein
HCC: Hepatocellular carcinoma
ICGC: International Cancer Genome Consortium
SOX: Sry-related high-mobility groupbox
TCGA: The cancer genome atlas
