# Supplementary material for: Development of an oncogenic dedifferentiation SOX signature with prognostic significance in hepatocellular carcinoma
Source: BMC Cancer. 2019 Aug 28;19:851. doi: 10.1186/s12885-019-6041-2 (PMC6714407; doi:10.1186/s12885-019-6041-2)
Supplement: Supplementary file 1 — Table S1. Clinical characteristics of the patients. (DOCX 24 kb) [file 12885_2019_6041_MOESM1_ESM.docx]

|  | TCGA  cohort I | TCGA  cohort II | LIRI-JP  cohort |
| --- | --- | --- | --- |
| No. of patients | 189 | 188 | 231 |
| Gender, No. (%) |  |  |  |
| Male | 123 (65.1) | 132 (70.2) | 171 (73.7) |
| Female | 66 (34.9) | 56 (29.8) | 60 (26.3) |
| Tumor Stage, No. (%) |  |  |  |
| I | 96 (50.8) | 81 (43.1) | 36 (15.5) |
| II | 37 (19.6) | 51 (27.1) | 106 (45.7) |
| III | 43 (22.8) | 43 (22.9) | 70 (30.6) |
| IV | 1 (0.5) | 1 (0.5) | 19 (8.2) |
| Tumor Grade, No. (%) |  |  |  |
| G1 | 21 (11.1) | 34 (18.1) | NA |
| G2 | 91 (48.1) | 89 (47.3) | NA |
| G3 | 68 (36.0) | 56 (29.8) | NA |
| G4 | 6 (3.2) | 7 (3.7) | NA |

**Additional file 1: Table S1. Clinical characteristics of the patients**
