# Supplementary material for: Development of an oncogenic dedifferentiation SOX signature with prognostic significance in hepatocellular carcinoma
Source: BMC Cancer. 2019 Aug 28;19:851. doi: 10.1186/s12885-019-6041-2 (PMC6714407; doi:10.1186/s12885-019-6041-2)
Supplement: Supplementary file 2 — Table S2. Sequences of primers used in qPCR. (DOCX 22 kb) [file 12885_2019_6041_MOESM2_ESM.docx]

**Additional File2 Table S2** **Sequences of primers used in qPCR.**

| Primer | Sequence (5’-3’) |
| --- | --- |
| qRT-SOX3-F | ACCAGGACCGTGTGAAACG |
| qRT- SOX3-R | CGTCGATGAATGGTCGCTTCT |
| qRT- SOX4-F | GACCTGCTCGACCTGAACC |
| qRT- SOX4-R | CCGGGCTCGAAGTTAAAATCC |
| qRT- SOX12-F | AAGAGGCCGATGAACGCATT |
| qRT- SOX12-R | TAGTCCGGGTAATCCGCCAT |
| qRT-18S-F | AACCCGTTGAACCCCATT |
| qRT-18S-R | CCATCCAATCGGTAGTAGCG |
| qRT- SOX14-F | CCACCCTGGGCTACCAGAA |
| qRT- SOX14-R | GCCAGTCTTGGTCATGCCT |
| qRT- SOX11-F | AGGATTTGGATTCGTTCAGCG |
| qRT- SOX11-R | AGGTCGGAGAAGTTCGCCT |
|  |  |
