# Supplementary figures and images for: Development of an oncogenic dedifferentiation SOX signature with prognostic significance in hepatocellular carcinoma
Source: BMC Cancer. 2019 Aug 28;19:851. doi: 10.1186/s12885-019-6041-2 (PMC6714407; doi:10.1186/s12885-019-6041-2)

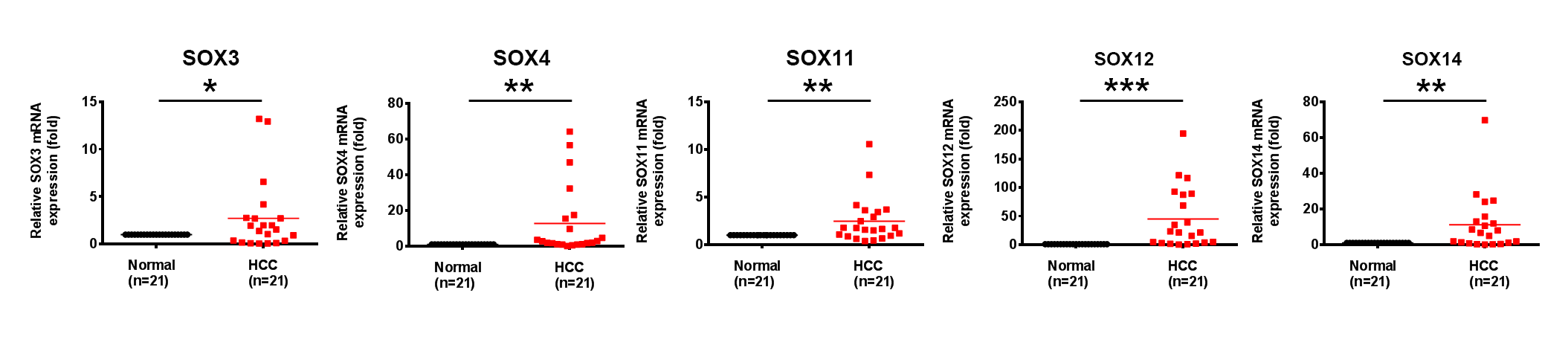

Supplement: Supplementary file 3 — Figure S1. Relative expression of SOX signature genes in paired HCC clinical samples. (TIF 3233 kb) [file 12885_2019_6041_MOESM3_ESM.tif]

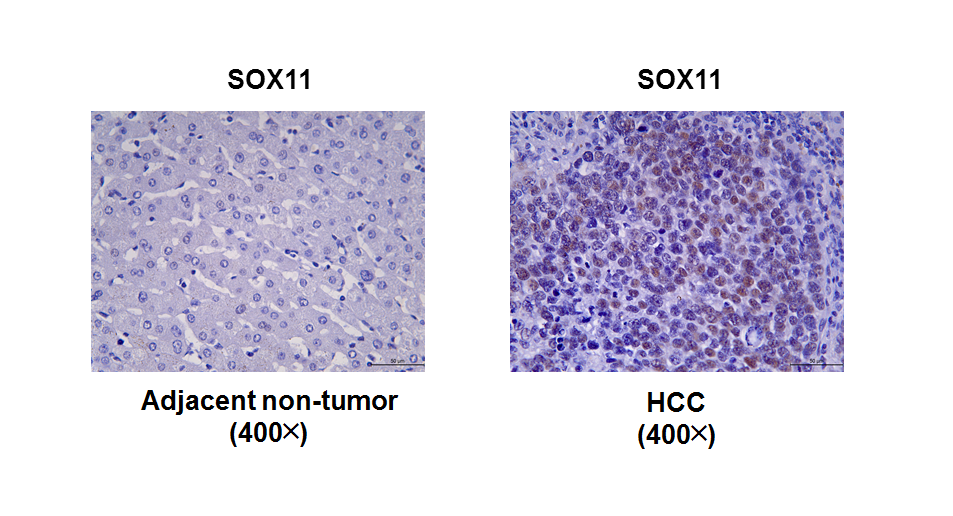

Supplement: Supplementary file 4 — Figure S2 Overexression of SOX 11 in paired HCC clinical tissues. (TIF 2043 kb) [file 12885_2019_6041_MOESM4_ESM.tif]
