# Supplementary material for: Development of an oncogenic dedifferentiation SOX signature with prognostic significance in hepatocellular carcinoma
Source: BMC Cancer. 2019 Aug 28;19:851. doi: 10.1186/s12885-019-6041-2 (PMC6714407; doi:10.1186/s12885-019-6041-2)
Supplement: Supplementary file 5 — Table S3 Predicted downstream targets of SOX signature genes. (DOCX 24 kb) [file 12885_2019_6041_MOESM5_ESM.docx]

**Additional file 5: Table S3. Predicted downstream targets of SOX signature genes**

| Number | Gene |
| --- | --- |
| 1 | XDH |
| 2 | DDB1 |
| 3 | ZEB1 |
| 4 | RPS6KA5 |
| 5 | GCLM |
| 6 | IDO2 |
| 7 | LTBR |
| 8 | KRCC1 |
| 9 | TNKS2 |
| 10 | HN1 |
| 11 | SLC25A24 |
| 12 | FAM84B |
| 13 | RFK |
| 14 | WIBG |
| 15 | NDE1 |
| 16 | ANXA2 |
| 17 | SH3PXD2B |
| 18 | SCG2 |
| 19 | GLIS3 |
| 20 | LRRC70 |
| 21 | TBL1XR1 |
| 22 | CDC45 |
| 23 | PPP1CB |
| 24 | KIAA0895 |
| 25 | NONO |
| 26 | ERMN |
| 27 | ABCB11 |
| 28 | TRIM5 |
| 29 | SAV1 |
| 30 | YPEL2 |
| 31 | ZNF248 |
| 32 | IRF2BP2 |
| 33 | PELO |
| 34 | HDDC3 |
| 35 | UFD1L |
| 36 | DCUN1D3 |
| 37 | RHAG |
| 38 | ANXA2R |
| 39 | KLHL4 |
| 40 | DNMBP |
| 41 | FLRT2 |
| 42 | C7orf25 |
| 43 | MAP4K4 |
| 44 | SNED1 |
| 45 | SDCBP2 |
| 46 | SUOX |
| 47 | ATF7IP2 |
| 48 | CYP39A1 |
| 49 | SERPINB9 |
| 50 | RIN2 |
| 51 | FAM162B |
| 52 | PERP |
| 53 | ATP1A1 |
| 54 | TMEM212 |
| 55 | MEIS1 |
| 56 | TUBA1A |
| 57 | MEMO1 |
| 58 | NFKBIZ |
| 59 | EFCAB5 |
| 60 | CTSK |
| 61 | AP2B1 |
| 62 | XPOT |
| 63 | LIMS1 |
| 64 | MFAP3L |
| 65 | PI3 |
| 66 | STAT1 |
| 67 | TEDDM1 |
| 68 | HOXB4 |
| 69 | BAZ2B |
| 70 | SLC25A27 |
| 71 | AKR1B1 |
| 72 | MACC1 |
| 73 | DDX59 |
| 74 | SP3 |
| 75 | LAMP2 |
| 76 | BNIP3L |
| 77 | SREBF1 |
| 78 | NXT2 |
| 79 | ETV1 |
| 80 | ACTG1 |
| 81 | PHLDB2 |
| 82 | HNRNPA1 |
| 83 | PDLIM5 |
| 84 | SOX21 |
| 85 | LYRM1 |
| 86 | ADAMDEC1 |
| 87 | TMCO2 |
| 88 | NRP2 |
| 89 | SPTAN1 |
| 90 | AKIP1 |
| 91 | C14orf159 |
| 92 | DIEXF |
| 93 | TJP1 |
| 94 | GABPB1 |
| 95 | UBAP1 |
| 96 | GRAMD1A |
| 97 | NXPH4 |
| 98 | ANKRD16 |
| 99 | DAB2 |
| 100 | INHBA |
| 101 | PLCB1 |
| 102 | SEPP1 |
| 103 | PCF11 |
| 104 | FNBP4 |
| 105 | INTS9 |
| 106 | STK4 |
| 107 | SLC45A4 |
| 108 | BANP |
| 109 | CCDC88C |
| 110 | SRGAP3 |
| 111 | SCNN1A |
| 112 | ZNFX1 |
| 113 | IFNB1 |
| 114 | ETV6 |
| 115 | TMSB4X |
| 116 | UNC45A |
| 117 | TRIB2 |
| 118 | ZNF622 |
| 119 | CAPNS2 |
| 120 | NFIA |
| 121 | PPP1R15B |
| 122 | CCNE1 |
| 123 | TM4SF4 |
| 124 | ST8SIA4 |
| 125 | CCAR1 |
| 126 | ADH5 |
| 127 | RFX3 |
| 128 | TMEM156 |
| 129 | DUSP5 |
| 130 | CDC42BPA |
| 131 | USP32 |
| 132 | NFIL3 |
| 133 | C8orf86 |
| 134 | KCNJ6 |
| 135 | LYPD3 |
| 136 | MELK |
| 137 | HMBOX1 |
| 138 | IL1RAPL1 |
| 139 | TFB1M |
| 140 | TLE3 |
| 141 | ADHFE1 |
| 142 | CHD2 |
| 143 | FZD5 |
| 144 | HOXC10 |
| 145 | MEOX2 |
| 146 | THBS1 |
| 147 | HIST1H2AC |
| 148 | DGKA |
| 149 | ALKBH5 |
| 150 | DAK |
| 151 | KLHL5 |
| 152 | ZNF503 |
| 153 | ZDHHC2 |
| 154 | MBNL2 |
| 155 | SCOC |
| 156 | TBX5 |
| 157 | DCBLD2 |
| 158 | RBM24 |
| 159 | KIF11 |
| 160 | ARNTL2 |
| 161 | TTLL7 |
| 162 | REG1B |
| 163 | HOXA10 |
| 164 | SLC38A2 |
| 165 | EBF3 |
| 166 | CDC42EP2 |
| 167 | RRS1 |
| 168 | ZSWIM6 |
| 169 | IGFL2 |
| 170 | IL6 |
| 171 | CREB3L2 |
| 172 | ZNF238 |
| 173 | WIPI1 |
| 174 | PRSS23 |
| 175 | S100A10 |
| 176 | TDGF1 |
| 177 | RGAG1 |
| 178 | NRCAM |
| 179 | ENC1 |
| 180 | KIAA0430 |
| 181 | SLFN5 |
| 182 | TDO2 |
| 183 | CBX5 |
| 184 | UBC |
| 185 | IGF2BP3 |
| 186 | ANLN |
| 187 | C1orf216 |
| 188 | MCU |
| 189 | PLD1 |
| 190 | RARB |
| 191 | IL1RL2 |
| 192 | LANCL2 |
| 193 | PCBP1 |
| 194 | COL5A2 |
| 195 | MED25 |
| 196 | RASAL3 |
| 197 | ZCCHC10 |
| 198 | TOX |
| 199 | SH2D3C |
| 200 | BCL6 |
| 201 | PFN4 |
| 202 | FAM228B |
| 203 | NNMT |
| 204 | NR2F2 |
| 205 | PTTG1IP |
| 206 | FAM126A |
| 207 | C1QTNF9B |
| 208 | HOXA2 |
| 209 | CD99L2 |
| 210 | SEC24B |
| 211 | SH3BP5 |
| 212 | IARS |
| 213 | MED13 |
| 214 | ST5 |
| 215 | MLL5 |
| 216 | TMEM123 |
| 217 | GDE1 |
| 218 | ADAM17 |
| 219 | CDKN2C |
| 220 | TMEM107 |
| 221 | AMIGO2 |
| 222 | SSH2 |
| 223 | TRIM22 |
| 224 | FOXC1 |
| 225 | CLEC2B |
| 226 | HOXB3 |
| 227 | EMP1 |
| 228 | ITGAV |
| 229 | DNAJA1 |
| 230 | CRIM1 |
| 231 | PNRC1 |
| 232 | AIP |
| 233 | PTPRK |
| 234 | SPTLC2 |
| 235 | ARRDC3 |
| 236 | JARID2 |
| 237 | GOLIM4 |
| 238 | RHOBTB3 |
| 239 | SMARCA2 |
| 240 | BLOC1S6 |
| 241 | PLAC8 |
| 242 | FBXO18 |
| 243 | PHF10 |
| 244 | ATP2A2 |
| 245 | FAM110B |
